# Interpretable Log Contrasts for the Classification of Health Biomarkers: a New Approach to Balance Selection

**DOI:** 10.1128/mSystems.00230-19

**Published:** 2020-04-07

**Authors:** Thomas P. Quinn, Ionas Erb

**Affiliations:** a Independent Scientist, Geelong, Australia; b Centre for Genomic Regulation (CRG), The Barcelona Institute of Science and Technology, Barcelona, Spain; Dalhousie University

**Keywords:** balances, classification, coda, compositional data, log contrast, log ratio, machine learning, microbiome, prediction

## Abstract

Since the turn of the century, technological advances have made it possible to obtain the molecular profile of any tissue in a cost-effective manner. Among these advances are sophisticated high-throughput assays that measure the relative abundances of microorganisms, RNA molecules, and metabolites. While these data are most often collected to gain new insights into biological systems, they can also be used as biomarkers to create clinically useful diagnostic classifiers. How best to classify high-dimensional -omics data remains an area of active research. However, few explicitly model the relative nature of these data and instead rely on cumbersome normalizations. This report (i) emphasizes the relative nature of health biomarkers, (ii) discusses the literature surrounding the classification of relative data, and (iii) benchmarks how different transformations perform for regularized logistic regression across multiple biomarker types. We show how an interpretable set of log contrasts, called balances, can prepare data for classification. We propose a simple procedure, called discriminative balance analysis, to select groups of 2 and 3 bacteria that can together discriminate between experimental conditions. Discriminative balance analysis is a fast, accurate, and interpretable alternative to data normalization.

**IMPORTANCE** High-throughput sequencing provides an easy and cost-effective way to measure the relative abundance of bacteria in any environmental or biological sample. When these samples come from humans, the microbiome signatures can act as biomarkers for disease prediction. However, because bacterial abundance is measured as a composition, the data have unique properties that make conventional analyses inappropriate. To overcome this, analysts often use cumbersome normalizations. This article proposes an alternative method that identifies pairs and trios of bacteria whose stoichiometric presence can differentiate between diseased and nondiseased samples. By using interpretable log contrasts called balances, we developed an entirely normalization-free classification procedure that reduces the feature space and improves the interpretability, without sacrificing classifier performance.

## Abstract (precis)

High-throughput sequencing provides an easy and cost-effective way to measure the relative abundance of bacteria in any environmental or biological sample. When these samples come from humans, the microbiome signatures can act as biomarkers for disease prediction. However, because bacterial abundance is measured as a composition, the data have unique properties that make conventional analyses inappropriate. To overcome this, analysts often use cumbersome normalizations. This article proposes an alternative method that identifies pairs and trios of bacteria whose stoichiometric presence can differentiate between diseased and nondiseased samples. By using interpretable log contrasts called balances, we developed an entirely normalization-free classification procedure that reduces the feature space and improves the interpretability, without sacrificing classifier performance.

## INTRODUCTION

Many of the newest assays used in molecular research produce data that are relative in nature. This includes high-throughput sequencing (HTS), as used to quantify the presence of bacterial or gene species from environmental and biological samples. This also includes hyphenated chromatographic assays like liquid chromatography-mass spectrometry (LC-MS), as used to quantify the presence of proteins, lipids, or metabolites. HTS and LC-MS both generate high-dimensional data that can be used as health biomarkers to predict and surveil disease (1). They also both measure abundance by sampling from the total population. Consequently, the total number of molecules recorded for each sample is arbitrary, making these data compositional (2–8). Others have already demonstrated that compositionality confounds the routine application of univariate (9), correlation (10), and distance (11) measures. Since machine learning pipelines often rely on these measures, compositionality may impact the accuracy of classifiers trained on these data (2, 12).

Compositional data analyses tend to have one of three flavors depending on the transformation used. Although these transformations have technical differences, the choice between them will often depend on the desired interpretation. First, the “simple” log ratio approach uses a single reference to recast the data. Most commonly, the reference is the per-sample geometric mean (centered log ratio [CLR] transformation) or a single component (additive log ratio [ALR] transformation), but the geometric mean of interquartile range components (13) and of nonzero components (14) have also been proposed. After transformation, the analysis then proceeds as if the data were absolute, but with a caveat: the interpretation of the results depends on the reference used. Second, the “pragmatic approach” analyzes pairwise log ratios directly; this type of analysis has been used to score important genes (15) and gene pairs (16, 17), and to reduce the dimensionality of the data (17). This approach makes sense when the ratios themselves have some importance to the analyst. However, it presents a clear problem for the classification of high-dimensional data: ratios “explode” feature space from *D* features to D(D−1)/2 (pairs of) features, making the data even more high dimensional. Third, the “coordinate approach” uses an orthonormal basis to transform *D* components into *D* – 1 new variables via an isometric log ratio (ILR) transformation (18). One example of this approach is to define a set of “balances,” where each balance describes a log contrast between two groups of components (19–21). Balances have the formal appeal of the ILR transformation (i.e., orthogonality of the basis vectors and a full-rank covariance matrix) (19, 22) but can be more interpretable than general log contrasts because they are associated with successive bipartitions of the original feature set. These bipartitions are represented formally by a serial binary partition (SBP) matrix but can be more easily conceptualized as a dendrogram of the input variables. However, the utility of balances depends on having a desirable SBP (which must be manually curated or procedurally generated). One popular SBP decomposes the variance such that the first balance contains the most variance, the second balance the second most, and so on (23, 24). In microbiome research, authors have proposed using mean pH (25) or phylogeny (26, 27) to construct an SBP instead.

Several studies have applied supervised statistical learning to compositional data. Aitchison trained linear discriminant analysis (LDA) models on ALR-transformed data (28), as have others (29) (though LDA is now usually applied to ILR-transformed data [29, 30]). Generalized linear models, including logistic regression (LR), have also been used to classify compositional data (30, 31). However, both LDA and LR require at least as many samples as features, making them inappropriate for high-dimensional health biomarker data (though this limitation is mitigated by regularization, as used previously [32, 33] to classify compositions). Partial least squares (PLS), also suitable for high-dimensional data, has been applied to CLR-transformed data to predict continuous outcomes (34), while PLS discriminant analysis (PLS-DA) has been used to classify both CLR-transformed (35) and ILR-transformed (36) data. In microbiome research, a stepwise algorithm, implemented as selbal, was proposed to identify a single balance that performs well in classification and regression tasks (37). The last work highlights an advantage of balances: although ALR, CLR, and ILR transformations can facilitate statistical learning, balances can engineer the feature space into interpretable biomarker scores via balance selection. These biomarker scores are not unlike the *Firmicutes*-to-*Bacteroidetes* ratio previously found to be associated with obesity (38). In fact, one could think of balance selection as a way of finding important bacteria ratios in a more rigorous and general manner.

How best to classify high-dimensional compositional data remains an open question. We are not aware of any work that benchmarks compositional data transformations as they pertain to the classification of high-dimensional compositional data. In this study, we employed a statistically robust cross-validation scheme to evaluate how well regularized LR classifies health-related binary outcomes on 13 compositional data sets. Specifically, we benchmarked performance using features obtained from raw proportions, CLR-transformed data, balances, and selected balances. We used LR instead of other classifiers because the model weights can be interpreted directly as a measure of feature importance and because regression is a routine part of statistical inference. Our results show that the centered log ratio transformation, and all four balance procedures, outperforms raw proportions for the classification of health biomarker data. We also propose a new balance selection procedure, called discriminatory balance analysis, that offers a computationally efficient way to select important 2- and 3-part balances. These discriminant balances reduce the feature space and improve the interpretability without sacrificing classifier performance. In doing so, they also outperform a recently published balance selection method, selbal, in terms of runtime and classification accuracy.

## RESULTS AND DISCUSSION

### Choice in log ratio transformation does not impact performance.

**FIG 1:**
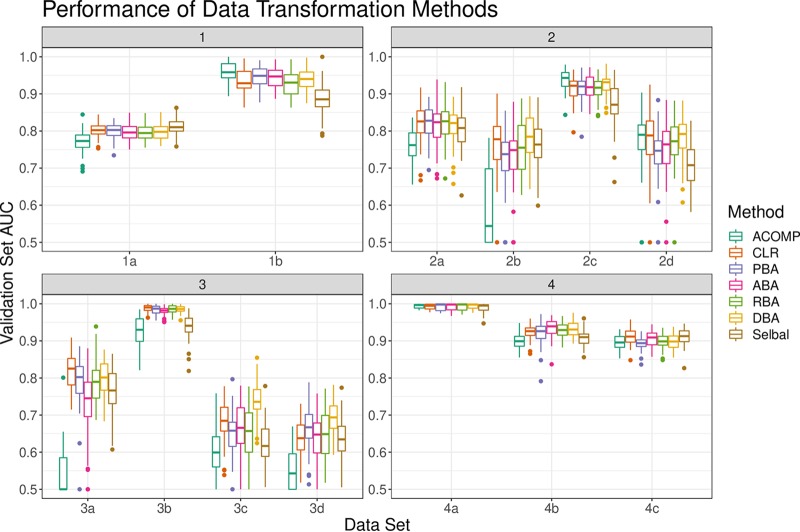
The distribution of validation set AUCs (*y* axis) for classifiers trained on closed or transformed data (*x* axis). Each validation set AUC describes a unique random training and validation set split. All classifiers are regularized logistic regression models, with *λ* tuned by training set cross-validation. Abbreviations: ACOMP, closed proportions; CLR, centered log ratio-transformed data; PBA, principal balances; ABA, anti-principal balances; RBA, random balances; DBA, discriminative balances.

Figure 1 shows the validation set areas under the receiver operating curves (AUCs) for binary classifiers trained on 13 data sets. In general, it can be seen that the centered log ratio transformation (CLR) and balance procedures (principal balance analysis [PBA], anti-principal balance analysis [ABA], random balance analysis [RBA], and discriminative balance analysis [DBA]) perform comparably. Although they all tend to outperform proportions (ACOMP), the proportions were more discriminative than the CLR for a few tests. This might occur when the closure bias itself confounds the predicted outcome.

Table 1 shows the median of the difference between data transformations (as computed with pairwise Wilcoxon rank sum tests across all 13 tests). Here, it can be seen that every transformation performs better than proportions. Also, all balance procedures tend to perform equally well, though DBA balances perform marginally better. Although selbal posts an impressive accuracy for only using a single balance, it is less accurate than using a set of all balances.

**TABLE 1:** Medians of the differences in performance between data transformation methods^*a*^

Method	Median of difference in performance of indicated method						
	selbal	PBA	ABA	RBA	DBA	ACOMP	CLR
selbal		0.0054 to 0.0324	0.0047 to 0.0318	0.0074 to 0.0339	0.019 to 0.045	−0.0290 to 0.0013	0.014 to 0.040
PBA	−0.0324 to −0.0054		−0.013 to 0.011	−0.0097 to 0.0138	0.0013 to 0.0248	−0.048 to −0.016	−0.003 to 0.020
ABA	−0.0318 to −0.0047	−0.011 to 0.013		−0.0092 to 0.0148	0.0018 to 0.0253	−0.045 to −0.015	−0.0026 to 0.0204
RBA	−0.0339 to −0.0074	−0.0138 to 0.0097	−0.0148 to 0.0092		−0.00061 to 0.02223	−0.048 to −0.017	−0.0048 to 0.0177
DBA	−0.045 to −0.019	−0.0248 to −0.0013	−0.0253 to −0.0018	−0.02223 to 0.00061		−0.060 to −0.029	−0.0144 to 0.0065
ACOMP	−0.0013 to 0.0290	0.016 to 0.048	0.015 to 0.045	0.017 to 0.048	0.029 to 0.060		0.024 to 0.054
CLR	−0.040 to −0.014	−0.020 to 0.003	−0.0204 to 0.0026	−0.0177 to 0.0048	−0.0065 to 0.0144	−0.054 to −0.024	

a Confidence intervals computed using pairwise Wilcoxon rank sum tests applied to 50 resamplings of 13 data sets. Abbreviations: ACOMP, closed proportions; CLR, centered log ratio-transformed data; PBA, principal balances; ABA, anti-principal balances; RBA, random balances; DBA, discriminative balances. This table corresponds to Fig. 1.

### DBA method selects predictive balances.

An advantage of using regularized logistic regression is that the model weights can be interpreted as a measure of feature importance. Even though the CLR and balances perform equally well, they imply different interpretations. Although the CLR data have one feature per component, the regularized weights do not describe the importance of that component. Rather, the CLR-based model weights describe the importance of that component relative to the sample mean. On the other hand, balances measure the log contrast between sets of components. Thus, the balance-based model weights describe the importance of those components directly.

For high-dimensional data, it can be challenging to interpret large balances. For example, the base of an SBP always contains one balance that comprises all variables. It may not be helpful in understanding the outcome to know that a log contrast involving all components is discriminative. On the other hand, smaller balances (i.e., those involving fewer components) might have a clearer meaning to the analyst. Here, we propose a new procedure, called discriminative balance analysis, to generate an SBP that makes the smallest balances most discriminative. This procedure can be used to engineer and select important balances prior to model building. Since the selected balances contain few parts, they are more easily interpreted.

**FIG 2:**
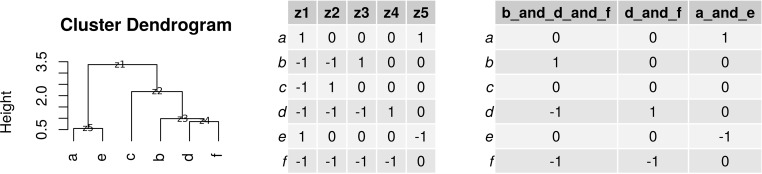
How a balance dendrogram relates to a serial binary partition (SBP) matrix. The left portion shows a dendrogram clustering the similarity between 6 components, where the first branch in the dendrogram refers to the first balance (i.e., *a* and *e* versus *c*, *b*, *d*, and *f*). The middle portion shows the corresponding SBP with 5 balances (columns) and the components involved in each log contrast (rows). The right portion shows the distal 2- and 3-part balances.

**FIG 3:**
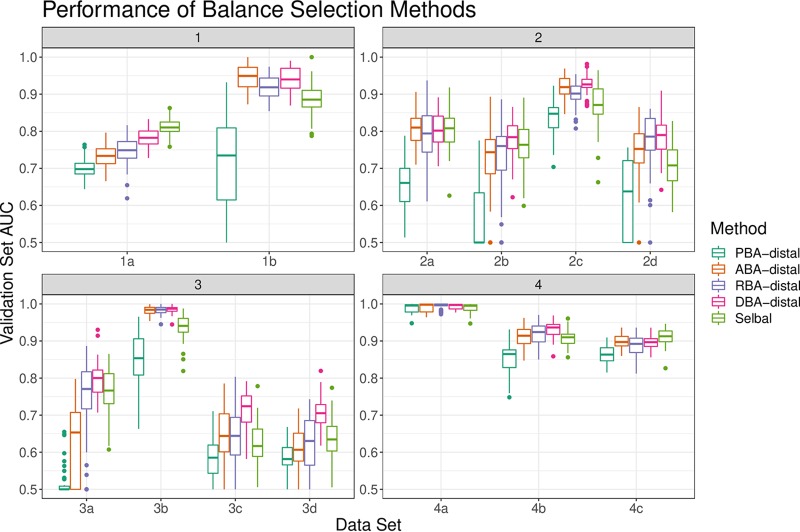
The distribution of validation set AUCs (*y* axis) for classifiers trained on selected balances (*x* axis). Each validation set AUC describes a unique random training and validation set split. All classifiers are regularized logistic regression models, with *λ* tuned by training set cross-validation. The appendix “-distal” indicates that only the 2-part and 3-part balances were used as features.

Conceptualizing the SBP as a tree, the largest balances are the “trunk” and the smallest balances are the “leaves” (Fig. 2). Since the SBP corresponds to an underlying orthonormal basis, we can treat each segment of the tree as its own variable. Figure 3 shows classification AUC using only the “distal leaf” balances (i.e., those with 2 or 3 parts). In principal balance analysis, the trunk contains the most variance, and the leaves contain the least. As expected, the distal PBA balances perform poorly. In anti-principal balance analysis, the trunk contains the least variance, and the leaves contain the most. As expected, the distal ABA balances outperform the distal PBA balances. In random balance analysis, balances are random, so the leaves might be discriminative by chance. As expected, the distal RBA balances have an average performance. In discriminative balance analysis, the trunk is least discriminative, and the leaves are the most. As expected, the distal DBA balances outperform both the PBA and ABA balances. Indeed, since DBA places the most discriminative balances distally, the distal DBA balances perform as well as all DBA balances (see Table 2 for 95% confidence interval).

**TABLE 2:** Medians of the differences in performance between balance selection methods^*a*^

Method	Median of difference in performance of indicated method					
	selbal	PBA-distal	ABA-distal	RBA-distal	DBA-distal	DBA
selbal		−0.125 to −0.091	−0.012 to 0.016	−0.0066 to 0.0203	0.016 to 0.042	0.019 to 0.045
PBA-distal	0.091 to 0.125		0.087 to 0.122	0.095 to 0.131	0.12 to 0.16	0.13 to 0.16
ABA-distal	−0.016 to 0.012	−0.122 to −0.087		−0.0082 to 0.0182	0.014 to 0.040	0.017 to 0.044
RBA-distal	−0.0203 to 0.0066	−0.131 to −0.095	−0.0182 to 0.0082		0.0082 to 0.0345	0.012 to 0.038
DBA-distal	−0.042 to −0.016	−0.16 to −0.12	−0.040 to −0.014	−0.0345 to −0.0082		−0.006 to 0.014
DBA	−0.045 to −0.019	−0.16 to −0.13	−0.044 to −0.017	−0.038 to −0.012	−0.014 to 0.006	

a Confidence intervals computed using pairwise Wilcoxon rank sum tests applied to 50 resamplings of 13 data sets. The appendix “-distal” indicates that only the 2-part and 3-part balances were used as features. This table corresponds to Fig. 3.

**FIG 4:**
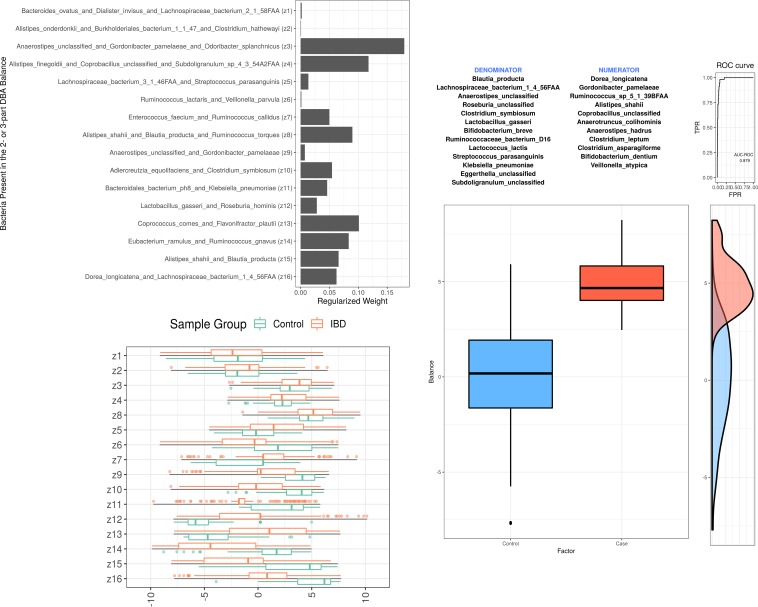
The most important distal DBA balances (left) compared with the results from selbal (right). In the top left portion are the regularized weights for each distal balance. In the bottom left portion is the distribution of samples for each balance irrespective of weight. The distal DBA classifier uses the weighted sum of these balances to make its prediction. In the right portion is the distribution of a single balance as selected by selbal. Many of the same variables are represented in both sets. DBA selects multiple simple balances instead of one complex balance. All panels generated using the 2a data set, comparing inflammatory bowel disease (in red) with healthy controls (in blue).

The DBA balances can be interpreted (and visualized) in an intuitive way. The 2-part balances can be visualized as a log ratio, while the 3-part balances can be visualized with a ternary diagram or as a log contrast. In Fig. 4, we compare the most important distal DBA balances (left) with the single discriminative balance found by selbal (right). It can be seen that many of the same variables are represented in both sets. However, DBA expresses the important variables via 2- and 3-part subsets that are, by definition of the SBP, grouped to be maximally discriminative. On the left side, it can be seen that balances with large regularized weights (top left) have log contrast scores that differentiate the groups (bottom left). Though selbal performs remarkably well in its ability to select a single discriminative balance, our results suggest that the distal DBA method outperforms selbal by ∼1 to 4% AUC (Table 2). Moreover, the distal DBA method is an order of magnitude faster than selbal, the latter of which must try multiple component combinations before finding the best log contrast (25 min versus 15 s for 1,000 features).

We cannot guarantee that these performance trends will hold for nonlinear classifiers like random forests or neural networks. However, a primary advantage of balances is that they allow for a clear interpretation of feature importance that is fully coherent for compositional data. If we do not first log ratio transform these relative data, then the predictive potential of any one feature will depend on all other features. This is because the relative abundances themselves all depend on each other. For example, given the composition [*a*, *b*, *c*], an increase in *c* will decrease both *a* and *b*, but the balance between *a* and *b* will not change. The use of nonlinear classifiers alone does not address this fundamental issue.

### DBA as a discriminant ordination.

**FIG 5:**
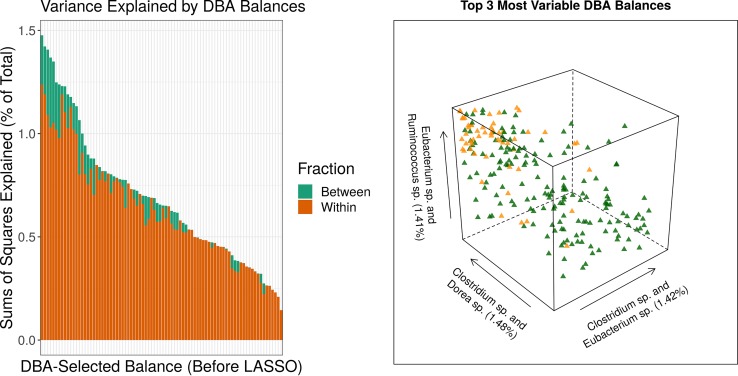
The amount of variance (as a percentage of the total) contained in each distal DBA balance (left), placed alongside a projection of the data across the top 3 most variable distal DBA balances (right). The sum of the between-group variance and the within-group variance equals the total variance. Good class separation is achieved using only 3 balances (each of which is proportional to a simple log ratio). Together, these 3 ratios contain 4.3% of the total variance and 13.8% of the total between-group variance. Both diagrams were generated using the 2a data set, comparing inflammatory bowel disease with healthy controls.

By using an orthonormal basis, balances represent the total variance in terms of new variables that allow us to quantify the variance contained in each discriminative balance. We can also break down the contained variance into its between-group and within-group fractions (as done by an analysis of variance [ANOVA]). The left side of Fig. 5 shows that a large fraction of the (log ratio) variance contained in the distal DBA balances is between-group variance. This is because clustering components by $\theta_{jj^{*}}$ will group together components whose pairwise log ratios describe only a small fraction of the within-group variance (i.e., a large fraction of between-group variance). Since the distal DBA balances are discriminative, we can use them to project a kind of discriminant ordination of the data. In other words, we can visualize the data along multiple interpretable axes (analogous to the axes in a discriminant analysis decomposing the variance between group means; however, for two groups, this would only give a single axis).

The right side of Fig. 5 shows good class separation using only 3 balances (each of which is actually a simple log ratio). From the left side, we know that these 3 axes contain 4.3% of the total variance and could likewise calculate that they contain 13.8% of the total between-group variance. Meanwhile, all distal DBA balances together account for 90.4% of the total between-group variance. Yet each one of these discriminant axes is fully interpretable, having no more than 3 parts. On the other hand, if the analyst cared less about interpretation and more about maximizing contained between-group variance, they could do a clustering of $1-\theta_{jj^{*}}$ and instead project the largest balance(s) thus obtained (in direct analogy to the principal balances heuristic described above).

A word of clarification about balances is in order. The term balances can be understood more strictly as the coordinates of an orthonormal basis of the sample space. Note that although this basis of the sample space is orthonormal, the balances themselves, when considered as vectors across samples, are not. Thus, discriminant balance variables will usually be correlated with each other.

### Summary.

This work benchmarks the performance of regularized logistic regression classifiers across 13 high-dimensional health biomarker data sets. Our results show that, on average, the centered log ratio and balances both outperform raw proportions in classification tasks. We also found that the serial binary partition (SBP) matrix used to generate the balances does not impact performance. However, the choice in SBP changes which balances are important for classification. In this report, we introduce a new SBP procedure that makes the most discriminative balances the smallest. This procedure, called discriminative balance analysis, offers a computationally efficient way to select important 2- and 3-part balances. These discriminant balances reduce the feature space and improve the interpretability without sacrificing classifier performance. In doing so, they also outperform a recently published balance selection method, selbal, in terms of runtime and classification accuracy. By using the distal DBA procedure, an analyst can quickly identify a set of highly interpretable bacteria ratios that best summarize the difference between their experimental conditions.

## MATERIALS AND METHODS

### Data acquisition.

We acquired data from 4 principal sources. Two gut microbiome data sets (originally published in references 39 and 40) were acquired from the selbal package (37). Two additional gut microbiome data sets (originally published in references 41 and 42) were acquired from the supplement to the work of Duvallet et al. (MicrobiomeHD database) (43). A fifth gut microbiome data set was acquired from the supplement to the work of Franzosa et al. (44).

The data of Schubert et al. (42) contained 3 classes comparingth hospital-acquired diarrhea (HAD) with community-acquired diarrhea (CAD) and healthy controls (HC). This data set was used in two tests: HAD versus CAD and HAD versus HC. The data of Baxter et al. (41) contained 3 classes comparing colorectal cancer (CRC) with adenoma (AC) and HC. This data set was also used in two tests: CRC versus AC and CRC versus HC. The data of Franzosa et al. (44) contained 3 classes comparing Crohn’s disease (CD) and ulcerative colitis (UC) with HC. This data set was also used in two tests: CD and UC versus HC and CD versus UC. Franzosa et al. also published gut metabolomic data for the same samples. These data were used for an additional two tests that paralleled the gut microbiome tests.

A sixth data set was acquired from The Cancer Genome Atlas (TCGA) (45) and contained microRNA expression for primary breast cancer (BRCA) samples and healthy controls (HC). We further labeled the BRCA samples using PAM50 subtypes retrieved from the supplement to reference 46. PAM50 uses a gene expression signature to assign an intrinsic subtype to the primary breast cancer sample: subtypes include luminal A (LumA), luminal B, HER2-enriched, Basal, and Normal-like. These data were used in three tests: any BRCA versus HC, HER2+ versus all other BRCA, and LumA-BRCA versus LumB-BRCA.

We selected these data because they are all publicly available and because they represent a range of difficult-to-classify data types (16S, metagenomic, metabolomic, and microRNA). All data are available for immediate use in subsequent benchmarks from https://doi.org/10.5281/zenodo.3378099.

### Feature extraction and zero handling.

Before training any models, features with too few counts were removed from the data. For the metabolomic and microRNA data sets, only features within the top decile of total abundance were included (this was done to reduce the feature space so that selbal became computationally tractable). For all data sets, features that contained zeros in more than 90% of samples were excluded (this was done to remove biomarkers that are not reliably present in the data). Finally, zeros were replaced using a simple multiplicative replacement strategy via the zCompositions package (47) (this was done because the Bayesian replacement strategy fails for heavily zero-laden data). Table 3 summarizes the tests used in this study.

**TABLE 3:** Data used to benchmark data transformation and balance selection methods^*a*^

Study code	Source	Type	Features	Group 1	Size	Group 2	Size	Median AUC
1a	selbal	16S	48	CD	662	HC	313	0.7924
1b	selbal	16S	60	MSM	73	Non-MSM	55	0.9359
2a	Franzosa et al.	Shotgun	153	IBD	164	HC	56	0.8166
2b	Franzosa et al.	Shotgun	158	CD	88	UC	76	0.7612
2c	Franzosa et al.	Metabolites	885	IBD	164	HC	56	0.9198
2d	Franzosa et al.	Metabolites	885	CD	88	UC	76	0.7703
3a	MicrobiomeHD	16S	278	Clostridioides difficile	93	Diarrhea	89	0.7431
3b	MicrobiomeHD	16S	610	Clostridioides difficile	93	HC	154	0.9821
3c	MicrobiomeHD	16S	1133	CRC	120	HC	172	0.6684
3d	MicrobiomeHD	16S	1302	CRC	120	Adenoma	198	0.6424
4a	TCGA	MicroRNA	188	Tumor	1078	Nontumor	104	0.9971
4b	TCGA	MicroRNA	188	Her2	77	Non-Her2	927	0.9149
4c	TCGA	MicroRNA	188	LumA	524	LumB	194	0.8974

a For reference, the last column also shows the grand median of all test set AUC scores. Abbreviations: CD, Crohn’s disease; HC, healthy control; MSM, men who have sex with men; UC, ulcerative colitis; IBD, inflammatory bowel disease; CRC, colorectal cancer.

### Data transformation.

Let us consider a data matrix with entries *x_ij_* which describe the relative abundance of j∈{1,…,D} components (as features) across i∈{1,…,N} compositions (as samples). Since the data studied are compositional, they can be expressed as a subcomposition of parts of the whole. The closure operation expresses the data so that the measurements for each sample sum to 1 (i.e., as proportions). The closed data are benchmarked in this study as the point of reference:

(1) $$\text{ACOMP}(x_{i})=\frac{\left[x_{i1},\ldots ,x_{\mathrm{iD}}\right]}{\sum_{j=1}^{D}x_{\mathrm{ij}}}$$

We also benchmark the popular centered log ratio (CLR) transformation:

(2) $$\text{CLR}(x_{i})=\text{log}\left(\frac{\left[x_{i1},\ldots ,x_{\mathrm{iD}}\right]}{\sqrt[{D}]{\prod_{j=1}^{D}x_{\mathrm{ij}}}}\right)$$

We also use the isometric log ratio (ILR) transformation to construct balances. Roughly speaking, balances are a way of combining the original features into new ones that better respect the geometry of the sample space. The most general way of doing so is in the form of a log-linear combination called a log contrast. A log contrast of a *D*-part composition $x_{i}$ is defined as $a_{1}\text{ log }x_{i1}+\ldots +a_{D}\text{ log }x_{\mathrm{iD}}$ with the constraint that $\sum_{j=1}^{D}a_{j}=0$. This constraint ensures scale invariance of the combination (i.e., a normalization factor of $x_{i}$ cancels). In the simplest case, a log contrast is just a log ratio.

Balances are a way of constructing simple log-contrasts that are relatively easy to interpret (18). This is done using a serial binary partition (SBP) matrix. The SBP matrix describes *D* – 1 log contrasts between the *D* parts. These log contrasts are special in that they have $a_{j}\in \{\frac{1}{d^{+}}\;\frac{-1}{d^{-}},0\}$. Here *d*^+^ and *d*^–^ refer to the number of positive and negative entries in a column of the SBP matrix (i.e., the number of parts in the numerator and denominator of the resulting log ratio). Such log contrasts thus have the form $\text{log}({\left(\prod_{j\in C^{+}}x_{\mathrm{ij}}\right)}^{1/d^{+}}/{\left(\prod_{k\in C^{-}}x_{\mathrm{ik}}\right)}^{1/d^{-}})$ where $C^{+}$ and $C^{-}$ are the sets of indices *j* with $a_{j}=\frac{1}{d^{+}}$ and $a_{j}=\frac{-1}{d^{-}}$, respectively. It is helpful to think of an SBP as a dendrogram tree, from which the *a_j_* can be derived (see Fig. 2 for an example SBP). A balance value is now computed for each sample *i* and each log contrast *z*:

(3) $$b_{\mathrm{iz}}=\sqrt{\frac{d_{z}^{+}d_{z}^{-}}{d_{z}^{+}+d_{z}^{-}}}\text{log}[\frac{{\left(\prod_{j\in C_{z}^{+}}x_{\mathrm{ij}}\right)}^{1/d_{z}^{+}}}{{\left(\prod_{k\in C_{z}^{-}}x_{\mathrm{ik}}\right)}^{1/d_{z}^{-}}}]$$

for the terms defined above. This particular form makes balances the coordinates of an orthonormal basis of the sample space (18). Although the formula seems elaborate, balances are easy to compute. For example, the 3-part balance *b* versus *d* and *f* (corresponding to *z*_3_ in Fig. 2), where for a given sample *i* we might have *x_ib_* = 3, *x_id_* = 4, and *x_if_* = 5, we would obtain the value $\sqrt{\frac{1\times 2}{1+2}}\text{log}\frac{3}{{\left(4\times 5\right)}^{1/2}}$.

### The serial binary partition matrix.

We benchmark four procedures for generating an SBP. In PBA, we approximate a set of principal balances by hierarchically clustering the log ratio variance matrix, *T*, describing the relationship between any two variables *j* and j* (see reference 24):

(4) $$T_{jj^{*}}=\text{var}[\text{log}\frac{x_{1j}}{x_{1j^{*}}},\ldots ,\text{log}\frac{x_{\mathrm{Nj}}}{x_{Nj^{*}}}]$$

Principal balances are analogous to principal components in that the first balance contains the most variance, the second balance the second most variance, and so on. Note that PBA only approximates the principal balances.

In ABA, we hierarchically cluster a new dissimilarity measure defined as the difference of the log ratio variance matrix from the maximum log ratio variance score: $\text{max}(T)-T_{jj^{*}}$. In RBA, we generate random SBPs using a custom algorithm that can make random binary trees (see balance::sbp.fromRandom for the source code). In DBA, we generate an SBP that maximizes the discriminative potential of the distal branches. This is done by hierarchically clustering the differential proportionality matrix, Θ, describing the relative contribution of the within-group log ratio variances ($T_{jj^{*}}^{1}$ and $T_{jj^{*}}^{2}$) to the total log ratio variance (see references 16 and 48):

(5) $$\theta_{jj^{*}}=\frac{N_{1}\;T_{jj^{*}}^{1}+N_{2}\;T_{jj^{*}}^{2}}{(N_{1}+N_{2})T_{jj^{*}}}$$

for groups sized *N*_1_ and *N*_2_. This matrix ranges from [0, 1], where 0 indicates that the two features have a maximally large difference in log ratio means between the two groups. Unlike the other SBP methods, the DBA method is supervised.

Note that the SBP is always constructed using the training set only. The balance “rule” is then applied to the validation set prior to model deployment. All SBP procedures are implemented in the balance package with the functions sbp.fromPBA, sbp.fromABA, sbp.fromRandom, and sbp.fromPropd (49). Differential proportionality analysis is implemented in the propr package (50) with the function propd. The code snippet below provides a minimally reproducible example for computing distal discriminant balances.

# how to get distal discriminant balances

install.packages(“balance”)

library(balance)

data(iris)

x <- iris[,1:4]

y <- iris[5,]

sbp <- sbp.fromADBA(x, y) *# get discriminant balances*

sbp <- sbp.subset(sbp) *# get distal balances only*

z <- balance.fromSBP(

 x = x, *# the data to recast*

 y = sbp *# the SBP to use*

)

### Classification pipeline.

In order to get a robust measure of performance, we repeat model training on 50 training sets randomly sampled from the data (with 33% set aside as a validation set). For each training set, we (i) transform features as described above, (ii) train a model on the transformed features, (iii) deploy the model on the withheld validation set, and (iv) calculate the area under the receiver operating curve (AUC). AUC is used because it is commonly reported in biological studies. Model splitting, transformation, training, and prediction are all handled by the high-throughput classification software exprso (51). By repeating this procedure 50 times, we can calculate the median performance and its range.

When using selbal, a generalized linear model is trained on a single balance (as described in reference 37). For all other transformations, a least absolute shrinkage and selection operator (LASSO) model is used to select features and fit the data simultaneously (via the glmnet package [52]). When using LASSO, *λ* is chosen procedurally by measuring 5-fold training set cross-validation accuracy over the series exp(seq(log(0.001), log(5), length.out = 100)) (i.e., from 0.001 to 5 in 100 exponential steps), with the best *λ* selected automatically by cv.glmnet.

We use regularized logistic regression because it is highly interpretable: the model weights can be interpreted directly as a kind of importance score.

### Availability of data and material.

All methods are available through open-source software maintained by us.
